# Islet cell autoantibodies in African patients with Type 1 and Type 2 diabetes in Dar es Salaam Tanzania: a cross sectional study

**DOI:** 10.1186/1740-2557-4-4

**Published:** 2007-10-27

**Authors:** JJK Lutale, H Thordarson, PI Holm, GE Eide, K Vetvik

**Affiliations:** 1 Institute of Medicine, Division of Haraldsplass Deaconal Hospital, University of Bergen, Norway; 2 Muhimbili University College of Health Sciences, Dar es Salaam, Tanzania; 3 Centre for International Health, University of Bergen, Bergen, Norway; 4 Department of Medicine, Haraldsplass Deaconal Hospital, Bergen, Norway; 5 Department of Medicine, Haukeland University Hospital, Bergen, Norway; 6 The Hormone Laboratory, Haukeland University Hospital, Bergen, Norway; 7 Centre for Clinical Research, Haukeland University Hospital and Section for Epidemiology and Medical Statistics, Department of Public Health and Primary Health Care, University of Bergen, Norway

## Abstract

**Background:**

The aim of the present study was to assess the occurrence of glutamic acid decarboxylase autoantibodies (GADA) and insulinoma antigen 2 autoantibodies (IA2A) among patients of African origin in Dar es Salaam, Tanzania and to compare the occurrence of autoimmune mediated Type 1 diabetes with findings previously reported from the same place and from other African diabetic populations.

**Methods:**

Two hundred and forty five patients from the diabetic clinic at Muhimbili Hospital were recruited for a cross sectional study. Patients were clinically classified into groups with Type 1 (T1D) and Type 2 diabetes (T2D); there were 94 patients with T1D and 151 with T2D. Autoantibodies for GAD and IA2 were measured with an assay based on radioligand binding. Fasting and random blood glucose, HbA1c, and C-peptide levels were also determined.

**Results:**

Of the patients with T1D, 28 (29.8%) were GADA positive and 20 (21.3%) were IA2A positive. The overall occurrence of any autoantibody was 42.6%. The GAD and IA2 autoantibodies were detected more frequently among patients with T1D than among patients with T2D (*P *< 0.001). A higher autoantibody prevalence was observed with combined GADA and IA2A measurements compared to individual autoantibody measurements; 40 (42.6%) patients with T1D versus 11 (7.3%) with T2D had at least one positive autoantibody titer. There was no correlation between duration of disease and detection of autoantibodies in patients with T1D. There was a strong association with family history of diabetes among the autoantibody positive versus autoantibody negative patients with T1D (p < 0.01).

**Conclusion:**

The prevalence of GAD and IA2 autoantibodies among African patients with T1D in Dar es Salaam was the same as that reported previously for South Africa and Ethiopia. It was much higher than the prevalence of islet cell autoantibodies (ICA) reported from the same clinic about 15 years ago. For unknown reasons the prevalence of pancreatic related autoantibodies in this African population is lower than the prevalence found among Caucasian populations.

## Background

For the past 30 years Type 1 diabetes mellitus (T1D) has been regarded as an autoimmune disease which leads to pancreatic β-cell destruction and absolute insulin deficiency [1]. However, evidence supporting the autoimmune component of T1D is inconsistent. It is unclear whether this is due to an inconsistency in techniques or an inconsistency arising from an underlying genetic variability in different populations. Studies in Caucasian populations have revealed an 80–90% occurrence of autoantibodies in patients newly diagnosed with T1D [2,3]. Recent reports from Asia have demonstrated an 83.3% – 90% occurrence of glutamic acid decarboxylase autoantibodies (GADA) in Japanese patients with T1D [4,5]. However earlier studies in the 1980's reported an occurrence as low as 7% of islet cell autoantibodies (ICA) in Japanese patients with T1D [6] and around a 40% occurrence in a mixed Asian population with T1D in Singapore [7,8].

There are relatively few studies regarding autoantibody prevalence in sub-Saharan Africa. During the 1980's scientific findings indicated a low occurrence of ICA ranging from 7–9% in patients with T1D from Nigeria and Tanzania [9,10]. On the other hand, studies during the same period reported an occurrence of ICA of 36% in patients with T1D of African origin from South Africa [11], and an occurrence of islet cell surface autoantibodies (ICSA) of 43% in patients from Ethiopia that required insulin to control diabetes [12]. A study from South Africa in 2002 [13] using a newer, simpler, and more precise method for detecting autoantibodies (by radioimmunoassay) identified GAD autoantibodies in 44% of patients with T1D and 2.5% of African patients with Type 2 diabetes (T2D). Studies in African Americans with newly diagnosed Type 1 diabetes also report a low ICA prevalence [14,15] compared with that reported in Caucasian populations with T1D. The studies from South Africa and Ethiopia suggest that autoimmunity plays a role in the pathogenesis of Type 1 diabetes in African patients as well. Because populations with Type 1 diabetes in Asia and Africa exhibit less than 50% prevalence of diabetes related autoantibodies, the American diabetes association (ADA) [16] and the World Health Organization [17] have sub-classified Type 1 diabetes into Types 1a and 1b, where Type 1a is autoimmune mediated, and Type 1b is non-autoimmune mediated, or idiopathic Type 1. There appear to be no significant clinical differences between patients with or without circulating autoantibodies [9,11,13]

Glutamic acid decarboxylase (GAD) and a protein tyrosine phosphatase-like molecule known as insulinoma antigen 2 (IA2) have been widely used as markers to describe the pathogenesis and the clinical course of Type 1 diabetes, though the nature of their involvement in the disease remains uncertain [3,18]. In Caucasians, about 10–15% of patients clinically diagnosed with T2D also have islet cell autoantibodies, especially GADA [19]. Currently, radioimmunoassays for GADA and IA2A are commonly used for assessing pancreatic islet cell autoantibodies. In the earlier study from Dar es Salaam, the occurrence of autoantibodies was not different in the IDDM and NIDDM patients [9]. This raises the question of whether some of the IDDM individuals were misclassified.

The aim of the present study was to assess the occurrence of autoantibodies in patients with Type 1 and Type 2 diabetes in Dar es Salaam using current techniques with GADA and IA2A and to compare these results with earlier findings from the same diabetic clinic, and also with the prevalence of autoantibodies reported in other studies from African countries.

## Methods

### Study Design and Patients

Two hundred and seventy one patients of African origin were enrolled between July 2003 and March 2004 in this cross-sectional study. All patients regularly attended the Diabetes Clinic of the Muhimbili National Hospital in Dar es Salaam. The Clinic does not differ in attendance from the other three municipal diabetes clinics in Dar es Salaam, as most of the patients attend both their municipal clinic and the Muhimbili Clinic. We therefore believe that patients attending the Clinic do not differ substantially from those attending the other clinics with regard to the type of diabetes, age, duration of disease, or sex distribution. The study was performed with the patients' written informed consent, according to the amended Declaration of Helsinki., and approved by the Scientific and Publication Committee of Muhimbili University College of Health Sciences and the Regional Committee for Medical Research Ethics of Western Norway.

### Data collection

A structured interview schedule was used to collect demographic information. Sociodemographic data included age at diagnosis, age at inclusion, gender, family history of diabetes, drinking and smoking habits, and the duration of diabetes. In cases of patient uncertainty the duration was calculated from the documented date of diagnosis. Other information was obtained from the patients' files, including the mode of presentation, type of treatment, and course of the disease.

### Clinical assessment

All patients were given a thorough physical examination. Body weight was measured to the nearest 0.5 kg and height to the nearest 0.5 cm. Body mass index (BMI) was calculated as weight (kg)/height (m^2^). Waist and hip circumference was measured and the waist-to-hip ratio calculated (WHR). Blood samples were collected (see below).

### Classification of diabetes

Patients were classified as Type 1 or Type 2 according to the clinical criteria recommended in the 1997 World Health Organisation [17] report. Classifications were based on age at diagnosis, mode of onset (acute versus insidious presentation), duration of disease, current treatment, BMI, waist-to-hip ratio, blood pressure, random or fasting glucose, HbA_1_c and urine ketones. A classification of Type 1 diabetes was defined by the following criteria: onset in patients aged 30 years or less, presentation of acute classical symptoms, and that required insulin therapy to control hyperglycaemia,. A classification of Type 1 diabetes was also designated to patients older than 30 years that required insulin treatment, lacked metabolic control, and were determined to be underweight. A classification of Type 2 diabetes was designated to patients who were older than 30 years at diagnosis and did not need insulin for metabolic control. A Type 2 classification was also designated to patients younger than 30 years who were obese and had diabetes for a long duration without requiring insulin treatment. Patients that did not clearly exhibit the clinical features of either Type were classified as undetermined and excluded from the present analysis.

### Blood sample collection and storage

A morning sample of 6 ml of venous blood was drawn from each subject and centrifuged within 2 hours of collection. The sera were frozen at -80°C and transported to Bergen in a dry ice-filled container that maintained the temperature between -50°C to -70°C. Samples reached their destination within 24 hours and were stored at -80°C.

### Blood glucose and HbA1c measurements

Capillary blood glucose was measured using a HemoCue AB glucose analyzer (Angelholm, Sweden). The accuracy range was 0–22 mmol/l; all readings above 22 mmol/l were displayed as ≥22 mmol/l.

HbA_1c _was measured immunochemically using a DCA 2000^®^+ (Bayer Corporation) [20]. The instrument was standardized against the DCCT method and was certified in 1996 [21].

### Autoantibody measurements

Radioligand assays were used for detecting both GADA and IA2A (CentAK^® ^anti-GAD; CentAK^® ^anti-IA2; manufactured by Medipan Diagnostics, Selchow, Germany). A highly purified human recombinant GADA-65 was labelled with 125-Iodine (^125^I). The tracer was added in excess to the samples and bound the GAD-65 autoantibodies. Likewise, a highly purified human recombinant IA2A (intracellular sample of IA2A) was labelled with ^125^I. The tracer was added in excess to the samples and bound the IA2 autoantibodies. All samples were run in duplicate and results were expressed in arbitrary units/ml. The radioactive signals (cpm) were measured with the Wallac 1470 Wizard^® ^Gamma counter machine (Wallac Oy, Finland). Samples were analyzed within 2 months of receipt.

The GADA and IA2A assays were evaluated in the first WHO Assay Proficiency Evaluation (Lab assay number 145) of the Diabetes Antibody Standardization Program (DASP) [22]. In this study the GADA assay had an adjusted median sensitivity of 80% and specificity of 90%, while the IA2A assay had a median sensitivity of 58% and specificity of 100%. In addition, the CentAK^® ^anti-GAD test was examined in the 4^th ^GADA Proficiency Study of the University of Louisiana (New Orleans, USA) in 1999, and displayed 100% specificity and sensitivity. The CentAK^® ^anti-IA2A was also examined in the 3^rd ^IA2 antibody Proficiency Study of the University of Louisiana, New Orleans, USA, and displayed 100% sensitivity and specificity. Furthermore, in a German comparative study of commercially available immunoradioactive assays, the Medipan CentAK^® ^GADA assays displayed 85% sensitivity and 100% specificity in newly diagnosed patients with T1D [23-26]. According to the respective instruction manuals, assay sensitivities (lower detection limits, corresponding to the 10% intra-assay and to the 20% inter-assay coefficients of variation in the precision profiles in the lower concentrations range) were 0.7 U/ml for the IA2 assay and 0.6 U/ml for the GADA assay. The reference values (normal ≤ 0.75 U/ml for the IA2 assay and <0.9 for the GADA assay) have been established in European populations, and were used in the studies mentioned above [23-26]. Mean coefficients of variance over the past year for control solutions with high and low titres respectively were 7.03% and 6.09% in our laboratory. To our knowledge no studies using CentAK^® ^antibody assays have been performed in ethnic groups other than Caucasians.

### C-peptide assays

The C-peptide assays were performed using the IMMULITE 2000 Automated Chemiluminescent Immunoassay Analyzer (Diagnostic Products Corporation kits of EURO/DPC Ltd., Flanders, NJ, USA). All samples were assayed in duplicate. The minimum detection limit was 0.003 nmol/l, with intra-assay variability of <9%. C-peptide concentrations were categorized in two groups: C-peptide negative and C-peptide positive. C-peptide-negative levels were those with either fasting C-peptide levels <0.3 nmol/l or glucose-stimulated [plasma glucose ≥10 mmol/l] C-peptide levels <0.6 nmol/l. The C-peptide-positive levels were those with fasting C-peptide levels ≥0.3 nmol/l or glucose-stimulated C-peptide levels ≥0.6 nmol/l.

### Statistical analysis

Distribution within groups was tested for skewness and distributions between groups was tested for statistically significant differences of standard deviations. Parametric and nonparametric tests were then used as appropriate. For continuous variables, either the unpaired t-test or the Mann-Whitney test (parametric and nonparametric tests) was performed. Differences between proportions were assessed by Fisher's exact test or by the chi-square test.

The correlation between disease duration and the occurrence of autoantibodies was determined with the Spearman nonparametric correlation test. Data were analyzed using SPSS version 14 for Windows. A *p-value *<0.05 was considered statistically significant.

## Results

### Sociodemographic characteristics of the study population

Two hundred and seventy one patients were enrolled; 54.3% were women. Eight patients did not complete the study and were excluded. Fourteen patients had presentations that could not clearly be classified as type 1 or type 2 diabetes, and they were excluded. Four patients who did not undergo autoantibody tests were also excluded. The final study population thus consisted of 151 patients with T2D and 94 patients with T1D. The sex distribution in the two groups was not different. The median overall duration of diabetes was 3.6 years (range 0–25). Patients with T2D had significantly longer median durations of diabetes than patients with T1D (4 versus 3 years, *p *< 0.001). Patients with T2D were significantly older and had higher BMI and WHR. In addition, a significantly (*p *< 0.001) higher proportion of patients with T2D had normal C-peptide levels compared to patients with T1D. Patients with T1D had a higher median HbA_1_c (13.7%) compared to that observed in patients with T2D (9.6%) (*p *< 0.001). (Table 1)

**Table 1:** Basic socio-demographic and biochemical characteristics of the patients with diabetes

**Variables**	**Type 1**	**Type 2**
Total *n *(%)	94 (38.4)	151 (61.6)
Female *n *(%) ^◊^	50 (53.2)	85 (56.2)
Age at diagnosis^◊◊ ^[median (min-max)] years	17 (4–60)	48 (20–79) ***
Diabetes duration^◊◊ ^[median (min-max)]years	3 (0–17)	4 (0–25)***
Family history of diabetes^◊ ^*n *(%)	42 (44.7)	67(45)
BMI^◊◊◊ ^[mean ± SD] kg/m^2^	19.5 ± 4.4	27.8 ± 4.7 ***
Waist-to-hip ratio ^◊◊◊ ^[mean ± SD]	0.87 ± (0.07	0.94 ± 0.1 ***
HbA_1c_^◊◊^[median (min-max)]%	13.7 (6->14)	9.6 (5.2->14)***
Normal C-peptide levels^◊# ^*n *(%)	7 (7.6)	96 (63.6) ***

*P < 0.05; **P < 0.01; ***P < 0.001.

^◊ ^Fisher's Exact Test ^◊◊ ^Mann-Whitney, ^◊◊◊ ^Unpaired t- test

^#^Normal C-peptide levels: fasting >0.3 nmol/l; random >0.6 nmol/l.

### GAD and IA2 autoantibody positivity

Autoantibodies were determined in 245 patients: 94 (38.4%) with T1D and 151 (61.6%) with T2D. Of the 245 patients, 36 (14.7%) had positive GADA levels (≥1 U/ml): 28 (78%) with T1D, and 8 (22%) with T2D. Twenty-three of the 245 (9.4%) patients had positive IA2A levels (≥1 U/ml): 20 (87%) with T1D and 3 (13%) with T2D. In the patients with T1D the frequencies of positive GAD (78%) and IA2 (87%) autoantibodies were not significantly different (*p *> 0.05). However, the detection of GAD and IA2 antibodies was significantly more frequent among patients with T1D than in patients with T2D (*p *< 0.0001).

Forty (43%) patients with T1D versus 11 (7.3%) patients with T2D had at least one autoantibody positive titer, and 8 (9%) patients with T1D versus no patients with T2D tested positive for both GADA and IA2A (Table 2). The detection of autoantibodies was not significantly influenced by gender in either class of diabetes.

**Table 2:** Prevalence of GADA and IA-2A in Type 1 and Type 2 diabetes

**Antibody status**	**Type 1 Diabetes N = 94** n(%)	**Type 2 Diabetes N = 151** n(%)
Both GADA- and IA-2A-positive	8(8.5)	0
GADA-positive only	20 (21.3)	8 (5.3)
IA-2A-positive only	12 (12.8)	3 (2)
Any antibody positive	40 (42.6)	11 (7.3)
Antibody negative	54 (57.4)	140 (92.7)

**Figure 1:**
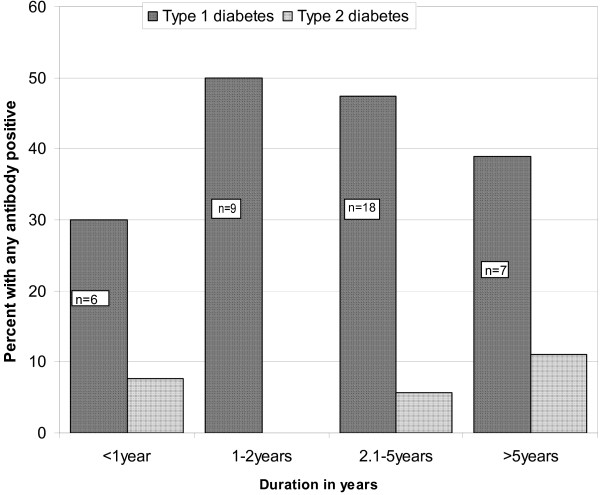
**Prevalence of any antibody in patients with different durations of diabetes (time intervals since diagnosis)**. In patients with T1D, the prevalence of any antibody was highest {*n *= 9, (50%)} in the 1–2 year interval of the disease, and thereafter dropped slightly. In patients with T2D the prevalence was lowest in the 1–2 year interval of the disease, and slightly increased thereafter. There was no inverse correlation between duration of disease and autoantibodies in the patients with T1D.

During the 1–2 year interval of the disease, patients with T1D exhibited the highest {*n *= 9, (50%)} frequency of detection of any autoantibodies; thereafter the frequency dropped slightly. However, there was no inverse correlation between the duration of disease and the frequency of detection of autoantibodies in the patients with T1D (Figure 1). In patients with T2D, the frequency of detection of any autoantibodies was lowest during the 1–2 year interval of the disease, and increased thereafter.

HbA_1_c, BMI, WHR, age at diagnosis, duration of disease and treatment with insulin were not significantly different between patients with T1D that were autoantibody positive or autoantibody negative. There was, however, a significantly higher proportion of patients with a family history of diabetes among those who were autoantibody positive compared with those who were autoantibody negative (*p *< 0.01, Table 3).

**Table 3:** Comparison of antibody-positive versus antibody-negative patients with T1D and their associated socio-demographic and biochemical characteristics

**Variables**	**Antibody status**	
	
	**Any antibody +ve**	**Antibody -ve**
Total *n *(%)	40 (43)	54 (57)
Female *n*^◊ ^(%)	23 (57.5)	27 (50)
Age at diagnosis^◊◊ ^[median (min-max)]years	18 (4–35)	17 (5–44)
Diabetes duration^◊◊ ^[median (min-max)] years	3.7 (0.2–17)	3.0 (0–14)
Positive family history of diabetes^◊ ^*n *(%)	24 (60)	18 (33.3)**
BMI ^◊◊◊ ^[mean ± SD] kg/m^2^	20.4 ± 5.4	19.2 ± 3.6
Waist-to-hip ratio^◊◊◊ ^[mean ± SD]	0.87 ± 0.09	0.87 ± 0.09
HbA_1_c ^◊◊ ^[median (min-max)] %	13.4 (7.4->14)	13.9 (5.9->14)
Normal C-peptide levels^◊# ^*n *(%)	2 (5.1)	5 (9.4)

*P < 0.05; **P < 0.01; ***P < 0.001.

^◊ ^Fisher's Exact Test, ^◊◊^Mann-Whitney, ^◊◊◊ ^Unpaired t test

^#^Normal C-peptide levels: fasting >0.3 nmol/l; random >0.6 nmol/l.

## Discussion

This study investigated the prevalence of GADA and IA2A in patients of African origin with diabetes in Dar es Salaam, Tanzania. The main finding from this study was a significantly higher prevalence of detecting any autoantibody (42.6%) among patients with T1D compared to the prevalence (7.3%) among patients with T2D. Eight (8.5%) patients with T1D and no patients with T2D had both GAD and IA2 autoantibodies. Our findings suggest that around 50% of patients with T1D have diabetes due to an autoimmune process. These findings contrast with those reported 15 years ago by McLarty et al from the same clinic; they found that the prevalence of ICA positive patients was not different among insulin-dependent and non-insulin-dependent patients [9]. They concluded that islet cell autoantibodies were not correlated to insulin-dependent diabetes. Another study from Nigeria used indirect immunofluorescence to measure islet cell autoantibodies; a method that is cumbersome and difficult to standardize [18]. They demonstrated a much lower frequency of autoantibodies than that reported here in patients with T1D [10]. However, our study is not directly comparable to those studies due to differences in the assays used, differences in sensitivity levels, and differences in the way patients were classified. On the other hand our findings are in accordance with the results from Ethiopian and South African studies [11-13]. Compared to other non-Caucasian ethnic populations with T1D, we found similar autoantibody prevalences. For instance, in a study from Saudi Arabia, patients with T1D were 54% positive for GADA and 27% positive for IA2A [27]. Additionally, in a study from Taiwan patients with T1D were 47% positive for GADA and 23.6% positive for IA2A [28].

To our knowledge, this is the first study to evaluate combined GAD and IA2 autoantibody measurements among African patients with diabetes. As reported in other studies, we found that measuring more than one type of diabetes-related autoantibody increased the likelihood of detecting Type 1 diabetes autoimmunity in a population [29-32].

Our findings suggest that around 50% of patients with T1D have the autoimmune mediated form of the disease (Type 1a diabetes, Figure 1). These results support the notion raised in previous reports from African studies regarding the existence of unknown disease mechanisms that operate in the other half the African population with T1D. We may, however, speculate that future studies in African populations are likely to uncover novel autoantibodies against beta cell components, demonstrating that the pathogenesis of Type 1b diabetes is autoimmune mediated as well.

Significantly more patients with T1D that were autoantibody positive had a positive family history of diabetes compared with those who were autoantibody negative; this is in accordance with the well-known notion that autoimmune diabetes has a genetic basis [33-35]. Some studies have shown disappearance of GADA and IA2A with time [36], while others have shown an increase of autoantibodies with time, up to a point, and still others have shown seroconversion to autoantibody positivity within the first few years of the disease after being negative at diagnosis [31]. About 75% of our patients had the disease for less than 4 years. Our results did not indicate any reduction in autoantibodies with time but rather showed a rise within the second year of the disease. Therefore it is unlikely that the low prevalence of autoantibody-positive patients in our study, compared with findings among Caucasians, might be explained by the disappearance of positive autoantibody titers as time passes.

## Conclusion

The prevalence of GADA and IA2 autoantibodies among African patients with T1D in Dar es Salaam was much higher than the prevalence of ICA previously reported from the same clinic about 15 years ago. However, the current findings are in accordance with reports from South Africa and Ethiopia. There is a strong association with family history among the autoantibody positive versus autoantibody negative patients with T1D. The prevalence of pancreatic related autoantibodies in this African population is considerably lower than prevalences found among Caucasians.

## Competing interests

None of the co-authors have any conflicts of interest to disclose in relation to the present study

## Authors' contributions

JJKL participated in the study conception and design, data collection and coordinated data management and analysis. Also wrote the initial and revisions of the manuscript. HT and KV participated in study conception and design, data analysis, and manuscript write up and reviews. PIH supervised laboratory hormone analysis and manuscript review. GEE participated in study design and data analysis. All authors read and approved the final manuscript.
